# Complex SUMO-1 Regulation of Cardiac Transcription Factor Nkx2-5

**DOI:** 10.1371/journal.pone.0024812

**Published:** 2011-09-12

**Authors:** Mauro W. Costa, Stella Lee, Milena B. Furtado, Li Xin, Duncan B. Sparrow, Camila G. Martinez, Sally L. Dunwoodie, Eleonora Kurtenbach, Tim Mohun, Nadia Rosenthal, Richard P. Harvey

**Affiliations:** 1 Developmental and Stem Cell Biology Division, Victor Chang Cardiac Research Institute, Darlinghurst, New South Wales, Australia; 2 Australian Regenerative Medicine Institute, Monash University, Clayton, Victoria, Australia; 3 Instituto de Biofísica Carlos Chagas Filho, Universidade Federal do Rio de Janeiro, Rio de Janeiro, Rio de Janeiro, Brazil; 4 St Vincent's Clinical School, Faculty of Medicine, University of New South Wales, Kensington, New South Wales, Australia; 5 Division of Developmental Biology, MRC National Institute for Medical Research, London, United Kingdom; French National Centre for Scientific Research, France

## Abstract

Reversible post-translational protein modifications such as SUMOylation add complexity to cardiac transcriptional regulation. The homeodomain transcription factor Nkx2-5/Csx is essential for heart specification and morphogenesis. It has been previously suggested that SUMOylation of lysine 51 (K51) of Nkx2-5 is essential for its DNA binding and transcriptional activation. Here, we confirm that SUMOylation strongly enhances Nkx2-5 transcriptional activity and that residue K51 of Nkx2-5 is a SUMOylation target. However, in a range of cultured cell lines we find that a point mutation of K51 to arginine (K51R) does not affect Nkx2-5 activity or DNA binding, suggesting the existence of additional Nkx2-5 SUMOylated residues. Using biochemical assays, we demonstrate that Nkx2-5 is SUMOylated on at least one additional site, and this is the predominant site in cardiac cells. The second site is either non-canonical or a “shifting” site, as mutation of predicted consensus sites and indeed every individual lysine in the context of the K51R mutation failed to impair Nkx2-5 transcriptional synergism with SUMO, or its nuclear localization and DNA binding. We also observe SUMOylation of Nkx2-5 cofactors, which may be critical to Nkx2-5 regulation. Our data reveal highly complex regulatory mechanisms driven by SUMOylation to modulate Nkx2-5 activity.

## Introduction

Post-translation modifications of transcriptional factors are required for strict spatial and temporal regulation of gene expression. Modifications such as phosphorylation, methylation and acetylation have long been established as essential regulators of signal transduction and other cellular processes. More recently, members of the Small Ubiquitin-related MOdifier (SUMO) family of proteins have been shown to play important roles in eukaryotic gene regulation [1]. SUMO is an 11-kDa polypeptide structurally related to ubiquitin, that can be covalently conjugated to lysine residues within target proteins at a consensus ψKXE site [where ψ is a large hydrophobic aminoacid and K is the site of SUMO conjugation [2]]. This modification is driven by an enzymatic cascade similar to ubiquitylation in structure but divergent with regards to its components and biological functions. While ubiquitylation has traditionally been associated with protein degradation, modifications by SUMO have been shown to affect protein-protein interactions, sub-nuclear localization and genome integrity in a variety of experimental models [3]. In the majority of cases, SUMOylation of transcriptional factors leads to transcriptional repression. This occurs via alteration of sub-nuclear localization [4], or by interaction with transcription co-repressors including HDACs, as shown for Elk-1, GATA1, GATA2 and SRF [5], [6], [7], [8]. Despite its usual repressive role, modifications by SUMO can also direct transcriptional activation, as is the case for HIF1-α, HSF1, NFAT-1 and p53 [9]. Interestingly, SUMOylation can activate transcription of muscle and cardiac-specific transcription factors, such as GATA4, myocardin and ERK5 [10], [11], [12]. In several instances, lysines targeted by SUMO are also sites for other post-translational modifications, such as acetylation, methylation and ubiquitylation. Thus, the nature of the modifier can lead to distinct functional outcomes, including changes in protein stability, sub-nuclear localization or transcriptional activity [1].

Nkx2-5/Csx is a homeobox transcription factor from the NK class expressed in cardiac progenitor cells and cardiomyocytes during embryonic development in all vertebrates. In the heart, this factor is essential for heart specification, spatial definition of chamber myocardium and formation and maintenance of elements of the conduction system [13], [14], [15], [16]. The *Nkx2-5* gene was initially identified by its high degree of homology to the *Drosophila tinman* gene, which is responsible for the formation of the dorsal vessel, the fly analogue of the heart [17]. In vertebrates, targeted deletion of *Nkx2-5* in embryos leads to severe impairment of cardiac morphogenesis and defects in ventricular and outflow tract development [18]. This phenotype is at least in part driven by loss of the repressive function of Nkx2-5 in regulating early proliferation of the secondary heart field and the Bmp2/Smad inductive pathway [19]. Highlighting its importance in cardiogenesis, more than 32 mutations in *NKX2-5* have been identified in patients with congenital heart disease, thus making this gene one of the most common single causes of such birth defects [20]. Recent data in mice have demonstrated that the wide range of clinical manifestations in Nkx2-5 mutants occurs through pleiotropic effects caused by genetic modifiers in the genome [21].

Here we show that over-expression of SUMO stimulates Nkx2-5 transcription up to 250-fold, but in disagreement with previous publications [22], [23], Nkx2-5 is SUMOylated *in vitro* in at least two dominant sites. One SUMOylation site corresponds to the previously identified K51 (21) while the other remains unidentified, occurring at a non-canonical or “shifting” site. We provide evidence that SUMOylation of Nkx2-5 and its cofactors synergistically activates transcription of specific promoters and potentially influences other post-translational modifications. Mutagenesis of K51 alone, or in the context of mutations in all other lysines individually, did not alter Nkx2-5 DNA binding, stability or nuclear localization.

## Methods

### Cell culture and transient transfections

HEK293T and COS-7 cell lines were obtained from ATCC (Rockville, MD). Cells were grown in Dulbecco's modified Eagle's medium (DMEM-GIBCO) supplemented with 10% (vol/vol) fetal bovine serum (GIBCO), maintained in a humidified 10% CO_2_ incubator at 37°C. Cells were seeded into 6-well plates to reach 70% confluence and were transiently transfected using Lipofectamine/Plus reagents (Invitrogen) as previously described [24]. For 6 well plates we used 0.25 µg of test plasmids, 0.2 µg of promoter construct (*pANFLuc*, encoding the atrial natriuretic factor reporter element; *Gja5*Luc, containing the −1190/+121 promoter region of mouse Cx40 gene [24], *Isl1Luc*, containing 1.5 Kb upstream of the transcription start site of Islet1; *Pitx2Luc*, containing 2.5 Kb upstream of the transcription start site of Pitx2 and *SM22αLuc*, with approximately 5 kb upstream of the transcription start site of smooth muscle SM22). Test plasmids were: pcDNA3-Nkx2-5, pcDNA3-Nkx2-5 I183P (Nkx2-5 mut), pFLAG-Nkx2-5, pMT3-HA-SUMO-1, pEM-Gata4, pEF FLAG-Tbx5, pcDNA3-Tbx20a [24], [25]. All pcDNA3-Nkx2-5 lysine to arginine mutants were generated by site directed mutagenesis (QuickChange Site-Mutagenesis Kit, Stratagene). SUMO-1ΔGG/Nkx2-5 fusion clones were obtained via the Gateway strategy (Invitrogen) and inserted in pcDNA3.2/V5-DEST (Invitrogen). pCGN-Nkx2-5 and pCGN-Nkx2-5K51R were described elsewhere (21). Cells were harvested after 48 hours. Transfections were performed in triplicates in at least 2 independent experiments. Expression patterns of wildtype and mutant Nkx2-5 in transfected cells were assessed using immunofluorescence microscopy and western blotting as previously described [25].

### Expression analysis

*In situ* hybridization methods were as described [25], [26]. Probes were generated by PCR, cloned into pGEMTeasy (Promega). To obtain sense and antisense transcripts, reactions were performed using T7 or SP6 RNA Polymerase (Roche). For qPCR, total RNA was isolated from tissues and cell lines using Trizol (Invitrogen) or RNAqueous 4PCR (Ambion). 1 µg of total RNA was treated with RQ1 DNase (Promega) and assayed for RT-PCR as previously described [24]. Realtime PCR was performed using specific mouse TaqMan Gene Expression Assay probes and assayed using the LightCycler 480 Real-Time PCR System (Roche).

### Co-immunoprecipitation and western blotting analysis

Lysates were prepared by harvesting transiently transfected HEK293T, COS-7 or HL-1 cells in Reporter lysis buffer, Passive lysis buffer (Promega) or CO-IP lysis buffer (50 mM Tris pH7.5; 15 mM EGTA; 100 mM NaCl; 0.1% Triton-X 100). For immunoprecipitation assays, cell lysates were incubated with anti-Flag M2 affinity gel (Sigma-Aldrich) or anti Nkx2-5 antibody (St. Cruz)/protein A sepharose (GE Healthcare) in CO-IP lysis buffer supplemented with 50 mM NEM (Sigma-Aldrich) to prevent de-SUMOylation, incubated at 4°C for 2 hours, and washed 3 times in PBS (with protease inhibitors and NEM). FLAG-tagged proteins were eluted by addition of 150 ng/µl 3×FLAG peptide (Sigma-Aldrich). For de-phosphorylation assays, cellular extracts were incubated with λPPA (New England Biolabs). Soluble extracts were subjected to immunoblotting with rabbit polyclonal anti-Nkx2-5 antibody (1∶600 dilution, Santa Cruz Biotechnology), mouse monoclonal anti-Nkx2-5 (1∶200 dilution, Abnova Corp.), mouse monoclonal anti-SUMO-1 (1∶1000 dilution, Developmental Studies Hybridoma Bank), mouse monoclonal anti-α-tubulin (1∶600 dilution, St. Cruz Biotechnology) and anti-HA antibodies (1∶4000 dilution, Sigma-Aldrich), anti-rabbit or anti-mouse IgG HRP-conjugated secondary antibodies (1∶4000 dilution, Amersham Biosciences), anti-rabbit Alexa Fluor 750 (1∶8000 dilution, Invitrogen) and anti-mouse Alexa Fluor 680 (1∶20000 dilution, Invitrogen). Membranes were developed using ECL-Plus detection reagent (Amersham Pharmacia) followed by X-ray film exposure or analysis using Odyssey Infrared Imaging System (LICOR Biosciences) [27].

### Immunohistochemistry

Wild type 9.5dpc embryos were fixed in 4% PFA, mounted in paraffin and sectioned. Immunohistochemistry was performed using rabbit polyclonal anti-Nkx2-5 antibody (1∶100 dilution, St. Cruz Biotechnology), mouse monoclonal anti-SUMO-1 (1∶100 Developmental Studies Hybridoma Bank), and anti-mouse IgG HRP-conjugated secondary antibody (1∶250 dilution, Amersham Biosciences), as previously described [27]. Staining was developed with DAB substrate (Vector Laboratories). Slides were mounted with VectaMount (Vector Laboratories) and photographed using a Zeiss Axiolab microscope at 20× magnification.

### Immunofluorescence

Transfected cells were fixed in 4% paraformaldehyde, washed in PBS, permeabilized with 0.1% Triton-X 100 and blocked with 10% goat serum. Cells were then incubated with primary rabbit polyclonal anti-Nkx2-5 antibody (1∶200 dilution, Santa Cruz Biotechnology), rabbit polyclonal anti-SUMO-1 (1∶100 dilution, Zymed Laboratories), and Alexa 555 anti-rabbit secondary antibody (1∶200 dilution, Invitrogen), and were mounted in Vectashield/DAPI (Vector Laboratories). Slides were examined with a Zeiss Axiolab fluorescence microscope at 40× magnification.

### Electrophoretic Mobility Shift Assay (EMSA)

*In vitro* translated [S^35^]-labelled Nkx2-5 (TNT Quick Coupled System, Promega) or nuclear extracts from transfected HEK293T or HeLa cells were mixed with binding buffer (25 mM Hepes pH 7.4; 75 mM NaCl; 1 mM MgCl_2_; 0.2 mM EDTA; 0.1% v/v NP-40; 1 mM DTT; 10 ng BSA; 200 ng poly dI/dC) and incubated for 10 min at room temperature. Annealed oligonucleotides (5 pM) were labelled using [γ^32^P] ATP and T4 polynucleotide kinase (New England Biolabs). 50000 cpm were added to each sample and incubated at room temperature for 20 min. 10 µl of the mixture was separated on a 5% PAGE gel using 0.5× TBE or 1× Tris-Glycine buffer, dried and subjected to autoradiography.

## Results

### SUMOylation machinery is expressed in the heart and potentially targets cardiac transcriptional factors

**Figure 1:**
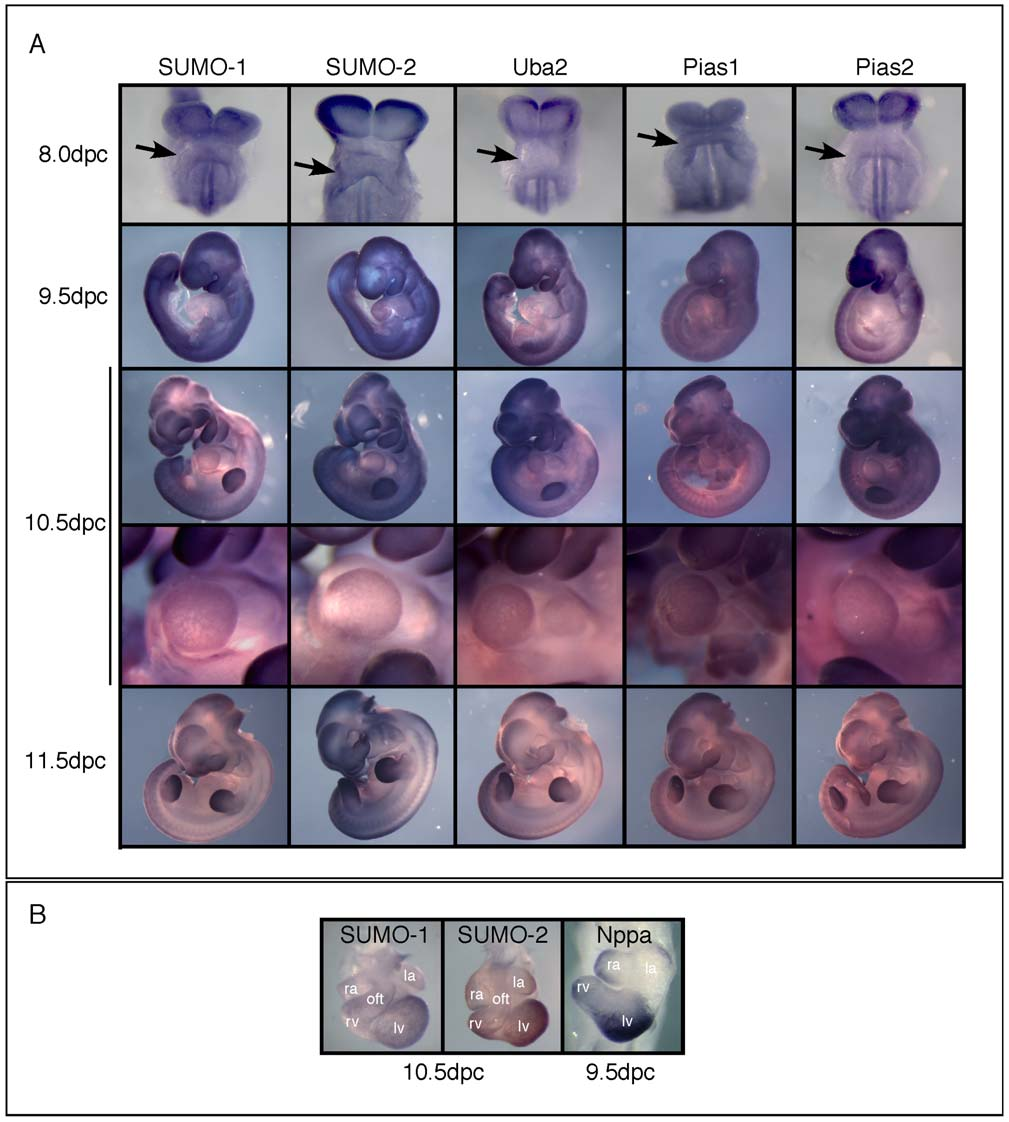
SUMOylation and cardiac embryonic development. (A) *In situ* hybridization analyses of SUMO pathway components at different developmental stages in the mouse (from 8.0 to 11.5 dpc, with ventral view at 8.0 dpc, and lateral view 9.5–11.5 dpc). Transcripts were seen in most embryonic regions, but enhanced expression could be noted in sites of extensive morphogenesis, such as the neural folds (8.0 dpc), branchial arches and limb buds (9.5 to 11.5 dpc). Arrows indicate cardiogenic regions at 8.0 dpc. (B) *In situ* hybridization revealed cardiac expression of SUMO-1 and SUMO-2, with stronger expression in the outer curvature, as demonstrated by *Nppa* transcripts. la – left atria; lv – left ventricle; rv – right ventricle; ra – right atria; oft – outflow tract.

SUMOylation of proteins is an essential process in cellular homeostasis. Ablation of key components of this pathway can lead to lethality and/or severe impairment of cellular functions in mice [9]. In an attempt to understand the role SUMOylation plays in cardiogenesis, we mapped expression of SUMO pathway components during heart morphogenesis. Whole-mount *in situ* hybridization experiments were performed in mouse embryos from 8.5 to 11.5 days post-coitum (dpc) (Figure 1A). mRNA transcripts for genes encoding SUMO-1, SUMO-2, Uba2, Pias1 and Pias2 were widespread although showed abundant enhancement at sites of intensive morphogenetic activity, such as the branchial arches and limb buds. The specificity of detection was demonstrated by the lack of signal using sense control probes (Figure S1). In cardiac development, enrichment of SUMO-1 and SUMO-2 mRNA was evident at the outer curvature of the developing heart tube, regions corresponding to the trabecular (working) chamber myocardium which specifically expresses a number of genes including that for atrial natriuretic factor (*Nppa*) (Figure 1B) [28]. This suggests a concomitant increase of components of the SUMOylation pathway in cardiac chamber regions undergoing proliferative growth and differentiation.

Consistent with SUMO expression in the heart, we had previously isolated a full length *Xenopus* SUMO-1 cDNA in a yeast two-hybrid (Y2H) screen of a *Xenopus* adult heart cDNA library, using full length *Xenopus* Nkx2-5 as a bait [29]. To map the interaction site, we created a series of amino- and carboxy-terminal deletions of *Xenopus* Nkx2-5, and tested for interaction with full length SUMO-1 in the Y2H assay (Figure S2). All tested deletions interacted with SUMO-1, suggesting that the protein contains several interaction sites in yeast. In contrast, the related homeodomain protein Nkx2-2 from *Xenopus* did not to interact with SUMO-1 in this assay. However, we failed to demonstrate direct protein-protein interaction between SUMO-1 and *Xenopus* Nkx2-5 using an *in vitro* glutathione S-transferase (GST) pull-down assay, perhaps because the interaction was weak out of its correct context, and/or that GAL4-SUMO-1 in fact became covalently linked to Nkx2-5 in the Y2H assay.

### Nkx2-5 is SUMOylated at lysine 51 and another non-canonical site

**Figure 2:**
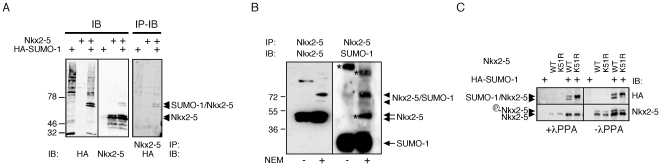
Nkx2-5 is SUMOylated in HEK293T and cardiac HL-1 cells. (A) Transiently transfected HEK293T cells expressing Nkx2-5 and HA-SUMO-1 proteins alone or simultaneously. Addition of HA-SUMO-1 led to the appearance of two extra bands by western blot, specific for both HA and Nkx2-5 antibodies. (B) Co-IP experiments performed in cardiac HL-1 cells with Nkx2-5 antibody display a similar pattern of SUMOylation (arrowheads) but with increased detection of the slow-migrating SUMOylated Nkx2-5 band. Stars (*) indicate SUMOylated proteins co-precipitated with Nkx2-5 antibodies. (C) Incubation of cellular extracts with λPPA caused the disappearance of the slower migrating band detected by Nkx2-5 antibodies (lower left panel), while no change in the pattern of migration of the Nkx2-5/SUMO-1 co-stained bands was observed (upper left panel).

To obtain stronger evidence for involvement of SUMOylation in cardiac transcriptional regulation, we turned to a cell culture system using the mouse homologue of Nkx2-5. HEK293T cells were transiently co-transfected with Nkx2-5 and/or HA-tagged SUMO-1. In the presence of SUMO-1, we were able to detect the appearance of two extra bands that reacted with both Nkx2-5 and HA-specific antibodies using western blotting, indicating that Nkx2-5 is indeed SUMOylated in this system (Figure 2A, left panels). These experiments were performed in the presence of N-ethylmaleimide (NEM), an inhibitor of SUMO-proteases, which cleaves off the SUMO moiety from modified proteins. The same bands were also detectable with an independent Nkx2-5 antibody (data not shown). Results were confirmed by immunoprecipitation (IP) of extracts from transfected cells with Nkx2-5 antibody, with subsequent western blotting using the HA antibody. The Nkx2-5 antibody also specifically precipitated the same two slowly migrating SUMOylated bands (Figure 2A, right panel). This pattern was also obtained in HeLa and COS-7 cell lines (Figure S3A, B), suggesting that the modification is cell-type independent. No bands of higher molecular weight (MW) were detected on these blots, indicating that either Nkx2-5 is modified at independent and exclusive sites, or that the levels of doubly SUMOylated Nkx2-5 fell below the level of reliable detection using this assay.

In order to confirm that the SUMO modification was present on native Nkx2-5 in cardiac cells we performed similar IP experiments in NEM-treated extracts from the atrial muscle cell line HL-1, which beats spontaneously in culture [33] and expresses endogenous Nkx2-5 and a host of other cardiac transcription factors important for heart development (data not shown). We were able to detect 2 endogenous Nkx2-5-SUMOylated bands running at the same MW as those detected in transfection studies that, as before, represented a minor proportion of the total Nkx2-5 pool. In this case there was a strong expression bias favouring the upper, more slowly-migrating species. These bands were detected after western blotting with antibodies for both Nkx2-5 and SUMO-1. At least two additional SUMO species were detected in the western blot with SUMO-1 antibody (Figure 2B), likely representing SUMOylated Nkx2-5 cofactors, or in the case of the larger band, a poly-SUMOylated species. A high MW Nkx2-5 or Nkx2-5-interacting species containing SUMO was also detected without NEM and diminished with NEM. The nature of these other complexes remains to be determined.

The presence of mono-SUMOylated bands migrating in different positions has been described for other proteins and may be due to the branched nature of modified proteins, or to additional post-translational modifications such as phosphorylation or acetylation [30], [31]. Previous studies have shown that Nkx2-5 can be phosphorylated at serine163 by casein kinase II [32] leading to a more slowly migrating band on PAGE (Figure 2C, lower right panel). To test whether phosphorylation of Nkx2-5 could lead to changes in SUMOylation pattern, extracts of transfected cells prepared in the presence of NEM were treated with λ Phosphatase (λPPA). This led to the disappearance of the more slowly migrating band of the main Nkx2-5 doublet (Figure 2C, lower left panel). However, no change in migration of the Nkx2-5/SUMO-1 bands was observed (Figure 2C, upper panels), indicating that the SUMOylation pattern was unrelated to the phosphorylation status of Nkx2-5. However, it remains possible that the phosphorylated form of Nkx2-5 cannot be SUMOylated *in vivo*.

The western blotting analysis performed thus far indicated that only a small portion of Nkx2-5 protein present in extracts from transfected cells was SUMOylated, even when extracts were prepared in the presence of the SUMO-protease inhibitor NEM. This is likely due to the highly transient nature of this modification and/or that only Nkx2-5 protein participating in active transcriptional complexes is SUMOylated. Electrophoretic mobility shift assay (EMSA) performed on NEM-treated extracts from HeLa cells transfected with Nkx2-5 plasmid, with or without the HA-SUMO plasmid, we were able to demonstrate that the small proportion of the total Nkx2-5 protein pool that was SUMOylated was able to bind DNA. This was indicated by the presence of minor supershifted bands in the presence of increasing concentrations of HA antibody (Figure S3C).

**Figure 3:**
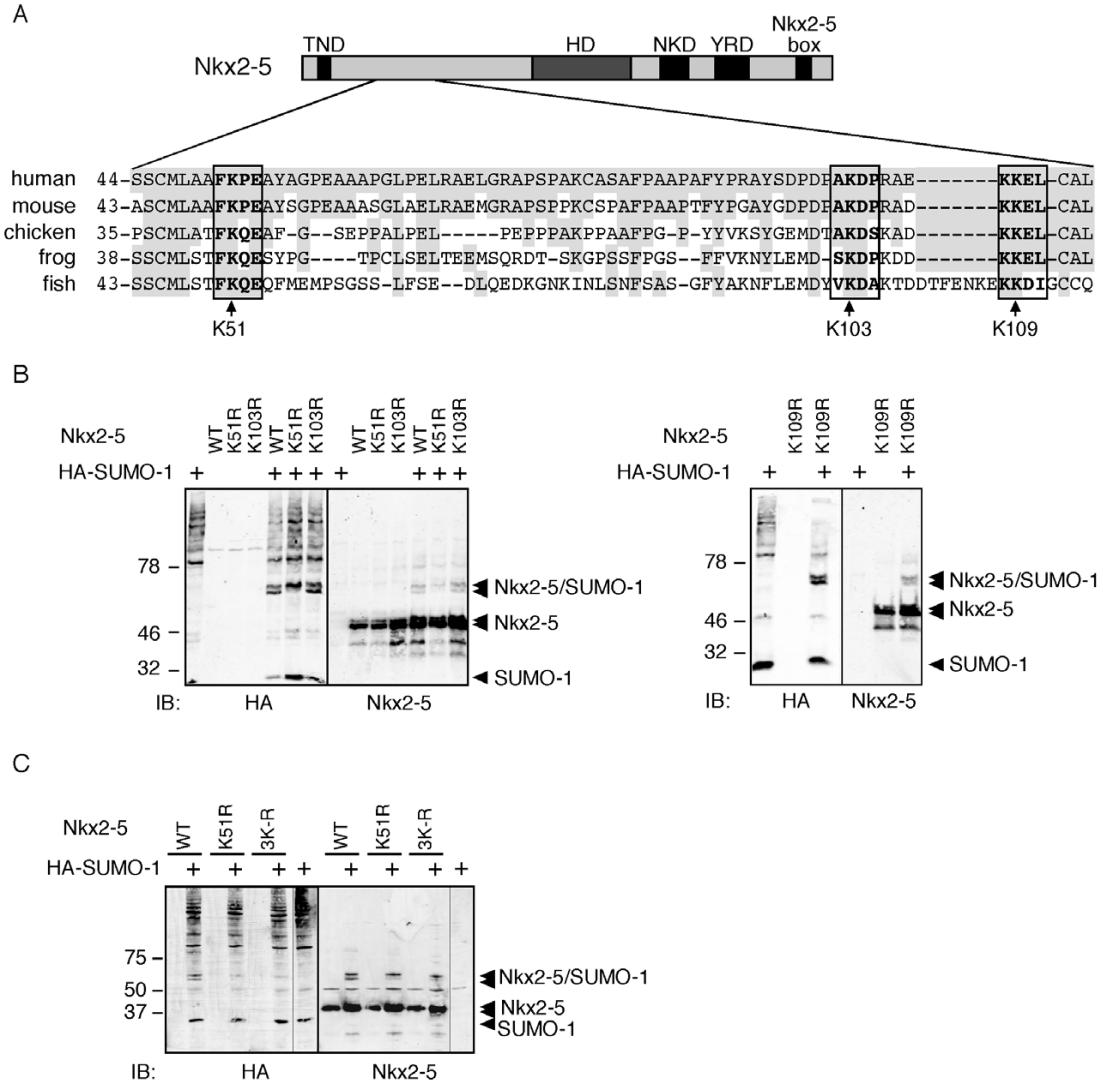
Identification of putative Nkx2-5 SUMOylation sites. (A) Nkx2-5 sequences from fish to humans were analyzed bioinformatically and canonical conserved sites were identified at the amino-terminal region of the protein. (B–C) HEK293T cells were co-transfected with HA-SUMO-1 and single putative SUMOylation site mutants (K51R, K103R, K109R or the triple mutant 3K–R), and the band pattern was assessed by western blot with antibodies for HA and Nkx2-5. Transfection of K51R caused the disappearance of one previously detected SUMOylated band, indicating that K51 is a site for SUMO modification. All other mutants had identical SUMOylation pattern to the wild-type protein, suggesting that those were not SUMOylated sites. TND, TN domain; HD, homeodomain; NKD, NK2 specific domain; YRD, tyrosine-rich domain.

To identify the SUMOylation sites present in Nkx2-5 we first used a bioinformatics approach (Sumoplot™ – www.abgent.com/tools/toSumoplot). Two sites, (K51 [aa50–54; FKPE] and K103 [aa102–105; AKDP]) in the amino-terminal region of the mouse protein were identified as potential SUMOylation sites, although only K51 had the signature sequence ψKXE (Figure 3A and Figure S4). K51 showed an almost perfect conservation from human to fish while K103 displayed higher evolutionary divergence. Our analysis further revealed two less-conserved SUMOylation sites in the amino-terminus at residues 108–111 (K109; KKEL; Fig. 3A) and 122–125 (K123; DKAE; Figure S4). In order to increase the confidence of this prediction we performed an analysis using another algorithm, SUMOsp2.0 [34]. This confirmed K51 as a major site and also identified K109 when less stringent parameters were applied.

To characterize which residues of Nkx2-5 were actually modified in cells, the core lysines K51, K103 and K109 were changed into arginine by site-directed mutagenesis. K-to-R mutations have been previously shown to abolish SUMOylation. Nkx2-5 K51R, K103R and K109R mutant constructs were transfected in HEK293T cells and SUMOylation profiles assessed by western blotting with antibodies for HA-tag and Nkx2-5. Mutation at residue K51 led to the disappearance of the faster migrating band (Figure 3B), indicating that Nkx2-5 is SUMOylated at this site. Dephosphorylation of Nkx2-5 with λPPA did not affect the gel pattern of K51R (Figure 3C). The same results were obtained in HeLa cells (data not shown). However, K103 and K109 mutants had identical SUMOylation patterns to the wild-type protein (Figures 3B). The persistence of a slower migrating band with mutation of K103 and K109 implies either the presence of an additional site for SUMOylation in Nkx2-5, or perhaps the ability of both of the closely spaced K103 and K109 sites to accept a SUMO modification depending on which is dominant and available (a “switching” site). In order to test this hypothesis, a triple mutant construct (3K–R) was generated (Figure 3C). However, the upper band was still detectable, confirming the presence of an unidentified non-consensus SUMOylation site in Nkx2-5.

### SUMOylation of Nkx2-5 leads to transcription activation in a promoter-specific manner

**Figure 4:**
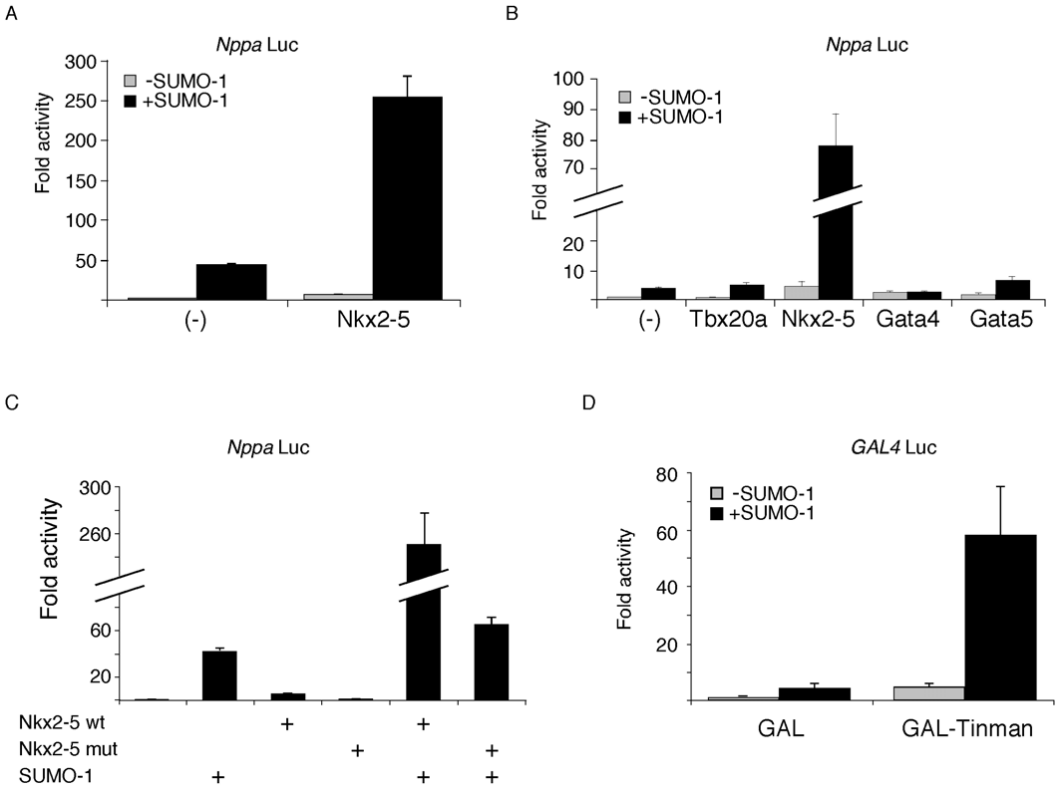
SUMOylation leads to synergistic activation of a cardiac-specific promoter by Nkx2-5. (A) HEK293T cells were transiently transfected with Nkx2-5, HA-SUMO-1 and the *Nppa* promoter. Activity is expressed as the fold-increase in luciferase expression. (B) No significant SUMO-1-mediated activation of the *Nppa* promoter was observed for other cardiac transcriptions factors (Tbx20a and GATA4/5), indicating that this effect was specific to Nkx2-5. (C) HEK293T were transiently transfected with wildtype Nkx2-5 or with a homeodomain point mutant that decreases DNA binding affinity. Addition of mutant protein abolishes SUMO-mediated activation. (D) The *Drosophila* homolog of Nkx2-5 gene, *tinman*, also displays SUMO-1-dependent transcriptional activity.

The presence of components of the SUMO pathway in the heart coupled to reports that SUMOylation is capable of regulating the activity of numerous transcription factors [35], [36] led us to test whether overexpression of SUMO-1 could affect Nkx2-5 activity on known cardiac target genes in HEK293T cells. As previously seen by our group and others, in such *in vitro* assays full length Nkx2-5 alone is normally a weak activator (around 4–5 fold) of the *Nppa* promoter, which carries an Nkx2-5 binding element (NKE) [24], [25], [37]. SUMO-1 showed an increase in background (Nkx2-5-independent) activity, but co-transfection of Nkx2-5 and SUMO-1 led to a robust increase in activation of the *Nppa* promoter. Stimulation was ∼50-fold compared with Nkx2-5 without SUMO-1, or ∼6-fold compared to SUMO-1 only (Figure 4A). This effect was dose dependent. Stimulation was also observed when CV-1 and COS-7 cells were used (Figure S5A, B).

The SUMO-1-mediated synergism observed for Nkx2-5 was specific, since it was not seen for other cardiac transcription factors that are capable of regulating the *Nppa* promoter region [24], [25] (Figure 4B). In order to test whether SUMO-mediated synergy requires Nkx2-5 binding to its DNA target site, we tested the ability of a functionally impaired mutant Nkx2-5, in which the isoleucine residue at position 183 was changed to proline (Nkx2-5mut), thus disrupting the helix-turn-helix fold of the homeodomain [38], to activate the *Nppa* promoter upon SUMOylation. Addition of Nkx2-5mut completely abolished SUMO-mediated activation, confirming that direct binding of Nkx2-5 to the *Nppa* promoter or to essential cofactors that interact directly with the homeodomain fold, is essential for SUMO-1-mediated synergy (Figure 4C). The *Drosophila* tinman protein, an Nkx2-5 orthologue, also displayed SUMO-1-dependent transcriptional activation, implying an ancient role for this modification in evolution of the cardiac regulatory network (Figure 4D). This activation occurred despite the lack of a homologous K51 site in the tinman amino-terminus, suggesting the presence of alternative sites for SUMOylation in tinman or the recruitment of other SUMOylated co-activators to transcription initiation sites.

**Figure 5:**
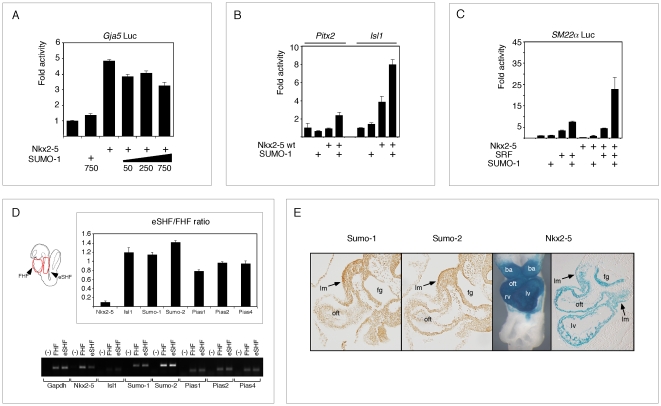
Synergism of SUMOylation with Nkx2-5 is promoter-specific. (A–C) SUMO-1 and Nkx2-5 failed to activate the cardiac promoters *Gja5* (A) and *Pitx2* (B) but could weakly activate *Isl1* (B) and *SM22* (C) promoters in HEK293T cells. (D) qPCR from First Heart Field (FHF) and trunk region enriched for Second Heart Field progenitors (eSHF) show presence of several SUMO components in both regions analysed. Regions used in this experimented are represented as red lines on E8.5 mouse embryo diagram (left). The relative levels on both regions were shown by non-saturated cycling PCR (30 cycles) followed by gel electrophoresis. (E) Immunohistochemistry of E9.5 embryos sections show Sumo-1 and Sumo-2 widely expressed and this pattern overlaps with Nkx2-5 expressing regions derived from Second Heart Field (SHF), including the outflow tract (oft) and lateral mesoderm (lm). lm – lateral mesoderm; oft – outflow tract; fg – foregut.

We also tested the ability of SUMO-1 to potentiate Nkx2-5-induced activation of other muscle and cardiac-specific promoters. SUMO-1 activity was promoter–specific, positively regulating *SM22α* and *Isl1*, but not significantly affecting the *Pitx2* promoter (Figure 5A–C). In contrast, SUMO-1 appeared to slightly repress Nkx2-5-dependent activation of the *Gja5* promoter. Isl1 is a marker of cardiac progenitor cells in the embryo and an important gene for deployment of second heart field (SHF) progenitors to the growing heart [19], [39], and our transfection data provide *in vitro* evidence that SUMOylation of Nkx2-5 and/or its cofactors directly regulates Isl1 promoter activity. Our whole-mount *in situ* hybridization experiments (Figure 1A) hinted that there was a spatial correlation between expression of SUMOylation pathway components and both the myocardium and its progenitor fields. In order to confirm this relationship, we dissected 8.0–8.5 dpc embryos and isolated linear/looping heart tubes, corresponding predominantly to descendants of the first heart field (FHF), and from adjacent trunk regions (second heart field enriched region – eSHF). Using qPCR (Figure 5D), we were able to demonstrate the presence of all analysed SUMO pathway mRNAs (encoding Isl1, SUMO-1, SUMO-2, Pias1, Pias2 and Pias4) in both heart and SHF. This was further confirmed by immunohistochemistry in 9.5dpc embryos, where Nkx2-5 and SUMO-1 showed co-localization in known SHF-derived embryonic regions - dorsal pericardial (splanchnic) and branchial arch (lateral) mesoderm (lm), and the outflow tract myocardium (oft) (Figure 5E). Together, this data suggests a role for SUMOylation in SHF transcriptional regulation.

### Mutagenesis of K51 SUMOylation site does not change nuclear localization and DNA binding affinity

**Figure 6:**
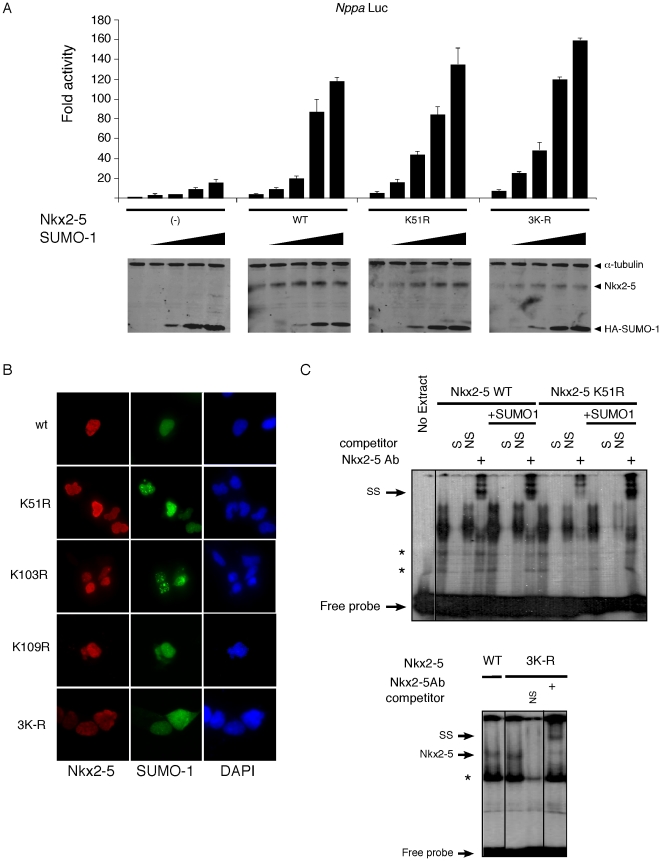
Mutagenesis of canonical SUMOylation site (K51) has no effect on Nkx2-5 transcriptional regulation. (A) SUMO-1 stimulates Nkx2-5-driven reporter activity in a dose-dependent manner, but SUMOylation-defective mutant proteins K51R and 3K-R had no effect on SUMO-dependent activation. Nkx2-5 and HA-SUMO1 protein levels were detected in cell extracts by western blot, with α-tubulin as loading control. (B) Immunofluorescence of transiently transfected HEK293T cells show normal nuclear localization of Nkx2-5 mutants K51R, K103R, K109R and 3K–R when compared to wildtype protein. (C) Nkx2-5 K51R binds to the NKE site with similar affinities to wildtype protein in HEK293T cells using EMSA. Overexpression of SUMO-1 leads to no detectable change in DNA affinities. S, specific oligonucleotide; NS, non-specific oligonucleotide; SS, supershift. Note that western blots were performed using extracts for Luciferase readings without NEM, therefore only the non-SUMOylated form of Nkx2-5 was detected.

Modifications by SUMOylation can lead to a broad range of effects in target proteins, including altered sub-nuclear localization, DNA affinity or interaction with other proteins [9], [40]. Mutagenesis of K51 or a combination of all 3 putative SUMOylation sites K51/103/109 (3K–R; see above) had no significant effect on Nkx2-5 SUMOylation-dependent transcriptional activity of the *Nppa* promoter (Figure 6A). In addition, mutation of K51 did not affect nuclear localization of co-overexpressed Nkx2-5 with SUMO-1 (Figure 6B). This was also observed when other lysines were mutated, or even for 3K–R (Figure 6B). To determine whether any of the K-to-R mutations in Nkx2-5 altered its DNA binding affinity, K51 and 3K–R were compared to wildtype Nkx2-5 using EMSA. No significant differences were detected between wildtype and mutant proteins with or without addition of SUMO-1 in either HEK293T or HeLa cell extracts (Figure 6C and data not shown).

**Figure 7:**
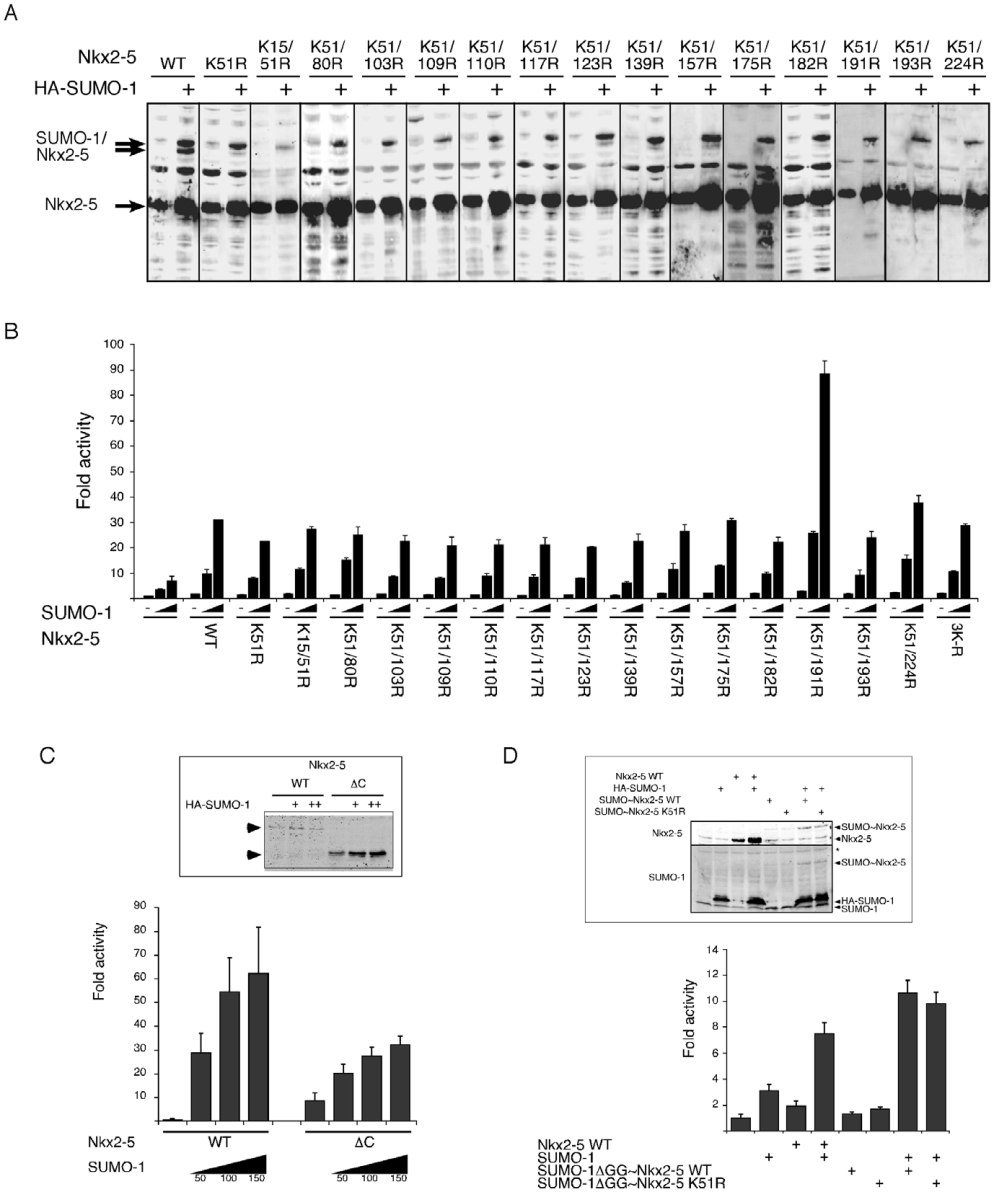
Complex regulation of Nkx2-5 activity by SUMOylation. (A–B) Mutagenesis of every lysine in Nkx2-5 in conjunction with K51R failed to change its SUMOylation pattern, as detected by western blot. (B) Most double mutant proteins were not capable of attenuating the activation exerted by SUMO-1 on Nkx2-5 when the *Nppa* promoter was used in co-transfection experiments performed in HEK293T cells. The exception was the double mutant K51/191R mutant that showed strong activation. (C) Deletion of the carboxi-terminal region of Nkx2-5 (ΔC) impaired SUMO-mediated activation on the *Nppa* promoter, despite enhancing Nkx2-5 stability in transient assays (inset). (D) A SUMO-1/Nkx2-5 fusion construct failed to elicit any activation of the *Nppa* promoter when compared to the Nkx2-5 WT construct alone, but activation was restored upon addition of exogenous HA-SUMO-1 constructs. Inset: western blot of extracts used for luciferase readings show presence of exogenously expressed Nkx-2-5 WT, HA-SUMO-1 and SUMO-1/Nkx2-5 fusions (SUMO-1∼Nkx2-5) WT and K51R. Endogenous SUMO-1 and possibly RanGAP1 (*) are also detected.

In an attempt to discover the second putative SUMOylation site, we changed every lysine in Nkx2-5 into arginine individually, in conjunction with K51. All double mutants still displayed the upper migrating band (Figure 7A) and were able to activate the *Nppa* promoter at similar or greater levels when compared to the wildtype construct (Figure 7B), indicating that none of these mutant combinations abolished the second SUMOylation site of Nkx2-5, or SUMO-stimulated transactivation. Interestingly, the double mutant K51/191 showed a significant increase in SUMO-mediated Nkx2-5 activation of *Nppa* in HEK293T cells (Figure 7B), suggesting that this residue is involved in a repressive activity, perhaps involving SUMOylation on chromatin at undetectable levels, or a distinct modification or cofactor interaction.

Deletion of the carboxi-terminal region of Nkx2-5 (ΔC) has previously been shown to significantly stimulate its transcriptional activity *in vitro*, and has led to the hypothesis that *in vivo*, the amino-terminal transactivation domain in Nkx2-5 is in some way masked by the carboxi-terminus or repressed by carboxi-terminal cofactor interactions [37]. While we also saw a significant stimulation of trans-activity in Nkx2-5ΔC, perhaps due to its increased stability, the SUMO-mediated activation seen with wt Nkx2-5 was significantly blunted (Figure 7C). This effect seemed independent of canonical SUMOylation status of Nkx2-5 in its carboxi-terminus, since the double mutant K51/224R (residue 224 is the only lysine present at the carboxi-terminal region) still showed strong SUMO-mediated activation of *Nppa* (Figure 7B). Our data suggest that SUMO-dependent activation could be mediated not so much by SUMOylation of Nkx2-5 itself, but by SUMOylation of its cofactors. In order to test this more directly, we generated a chimeric protein that fused SUMO-1 carrying a deleted deSUMOylation protease site (SUMO-1ΔGG) to the amino-terminal region of wild type or K51R Nkx2-5. Covalent linear attachment of SUMO to its target has been previously shown to mimic the activity of SUMO conjugation [41], [42], [43]. Despite being constitutively SUMOylated, the SUMO-1ΔGG-Nkx2-5 and SUMO-1ΔGG-Nkx2-5K51R constructs were not more active on the *Nppa* promoter when compared to transfection of Nkx2-5 alone (Figure 7D). However, both wildtype and K51R chimeric proteins retained the ability to be strongly augmented after co-trasfection of SUMO-1 plasmid. This occurred to levels similar to that of wt Nkx2-5, indicating functional integrity of the fusion constructs. In total, our results strongly suggest that SUMOylation of endogenous Nkx2-5 cofactors is a dominant component of SUMO-stimulated and Nkx2-5-dependent activation of the *Nppa* promoter in vitro (Figure 4C). We favour the view that Nkx2-5 activity is tightly regulated by a complex interplay between SUMOylation and ubiquitylation of both Nkx2-5 and its cofactors, as previously seen for other essential transcription factors [44].

## Discussion

Transcriptional activity during development must be tightly regulated in order to drive the dynamic changes that result in organ morphogenesis. This is in part achieved by tissue-specific transcription factors binding to gene promoter and enhancer regions, and recruitment of the general transcription machinery [45]. Transient post-translational modification of transcription factors allows a higher capacity for rapid changes in activity, and either binary or graded regulation of transcription. SUMOylation of proteins involved in transcriptional regulation has now been recognized as a widely used regulatory step in this process [36]. Here we show that SUMO modifiers are present in the heart and that enhancing SUMOylation within the cell by transfection of SUMO-1 plasmid dramatically enhances the activity of the cardiac transcription factor Nkx2-5, as previously shown for GATA4 and Myocardin [11], [12]. However, our findings suggest that regulation occurs through a complex mechanism involving direct SUMOylation and changes in the recruitment of SUMOylated co-activators.

Analysis of the expression pattern of components of the SUMOylation pathway by *in situ* hybridization in early mouse development revealed widespread expression of several components, reflecting the essential nature of this modification for normal cellular function. In contrast to ubiquitylation, the enzymatic pathway that leads to SUMOylation has a limited number of components, with only one E2 (Ubc9) and one E1 dimer (Aos1/Uba2) described so far [1], [46], [47]. In budding yeast, SUMO, E1 or E2 are required for cell survival, while depletion of E2 in worms, plants and mice results in embryonic death [1], [48], [49] and may also be involved in neurodegerative diseases like Huntington's disease (HD), spinocerebellar ataxias (SCA), Machado–Joseph disease, spinal and bulbar muscular atrophy (SBMA) and Aβ-amyloidogenesis [50]. Expression of SUMO-1, SUMO-2 and Uba2, and to a lesser extent Pias1/2, were moderately enriched in the outer curvature of the developing heart. This region, which differentiates into trabecular working myocardium, is demarcated by several chamber-specific transcription factors and other genes, including *Hand1*, *Cited1*, *Chisel/Smpx*, *Gja5* and *Nppa* [51]. The gene regulatory network established in chamber muscle during development is labile and requires signalling support provided by the endocardial layer [52]. The inner curvature (non-chamber myocardium) of the heart, in contrast, expresses Tbx2 and Tbx3, T-box transcription factors that inhibit chamber specification [53], [54]. Expression of cardiac compartment genes is thus under the strict control of a highly conserved pathway that includes Nkx2-5, Gata4 and multiple T-box factors [14], [16]. The ability of Nkx2-5 to interact with either activating (Tbx5 and Tbx20) or repressing (Tbx2, Tbx3) T-box factors allows specificity of control in different heart domains [13], [27]. Using an unbiased bioinformatic approach, Bauer et al. have shown that several transcriptional factors with activation/repression activities share conserved signatures for SUMOylation [55]. Nkx2-5 can also act as both activator and repressor, although it remains to be seen whether SUMOylation of Nkx2-5 participates in such changes [13], [19], [24], [56], [57].

SUMO-mediated enhancement of *Nppa* promoter in HEK293T cells requires Nkx2-5 direct interaction with its target sequence in the promoter. Mutation of one of the SUMOylation sites located at K51 did not abolish activity, or change nuclear localization or DNA binding. For this reason, we believe synergism between Nkx2-5 and SUMO-1 may be dependent on at least two SUMOylated residues. The potential second SUMOylation site is likely to be non-canonical in nature or “shifting”. Several extensions of the core SUMOylation site ψKXE have been proposed, including the PDSM (phosphorylation-dependent SUMOylation motif), the SC (synergy control motif) and the NDSM (negatively charged amino acid-dependent SUMOylation motif) [1]. We cannot discard the possibility of other SUMO modifications occurring at a site with no obvious signature, as seen for yeast PCNA and others [1], [46], [58]. We were not able to significantly affect gene activity upon mutation of all lysines in Nkx2-5 in the context of K51R. Therefore identification of the divergent site remains elusive. As noted, it is possible that mutation of one site leads to modification of another, as a buffering mechanism to retain protein activity.

SUMO-interacting motifs (SIMs) are transcription factor domains that have also been implicated in the regulation of promoter activity, as previously demonstrated for KLF4 and c-Myb [59], [60]. However, SIM motifs work independently of canonical SUMOylation sites. Recent data shows that a great part of the physiological outcomes of SUMOylation are mediated by co-factors that recognize SUMO-modified proteins through SIM motifs [47]. We demonstrated here that SUMO-1 is capable of interacting with *Xenopus* Nkx2-5 through multiple domains of the protein in yeast two-hybrid assays, but not with the closely related homeodomain factor Nkx2-2. Furthermore, deletion of Nkx2-5 C-terminal domain led to the attenuation of SUMO-mediated activation, independently of residue K224 as a candidate canonical SUMOylation site. Finally, our assays showed that a SUMO-1/Nkx2-5 fusion protein was not more active than Nkx2-5 alone, although like wt Nkx2-5 showed enhanced activity after augmentation of SUMO levels in the whole cell by transfection of SUMO-1 plasmid. Taken together, these results strongly suggest that Nkx2-5-associated proteins, potentially SUMOylated themselves, may be important for the synergistic activity exerted by SUMO over Nkx2-5.

Recently, Li et al. showed that the histone acetyl transferase (HAT) p300 interacts with the N-terminal region of Nkx2-5 [61], increasing its transcriptional activity. The C-terminal region of Nkx2-5 acts as a strong negative regulator *in vitro* [37], [62]. Deletion of this region increases Nkx2-5 activity, stability (this study) and affinity for p300. One of the most important mechanisms by which SUMOylation affects transcription is through changes in protein∶protein binding affinity [1], [9], [36]. Because the previously identified K51 SUMOylation site is localized whithin the region required for p300 interaction, it is tempting to speculate that SUMOylation might increase Nkx2-5 affinity for p300 or other HAT proteins and promote strict temporal and spatial regulation of target genes. In contrast, recruitment of histone deacetylases (HDACs) by SUMOylation is also a well-established mechanism of transcriptional repression [8], [63]. SUMO-regulation of these events may be important in vivo, although not rate-limiting for transcriptional assays after over-expression of components in vitro. SUMO-mediated activation of transcription is likely to be promoter specific, and indeed Nkx2-5 failed to elicit synergistic responses with SUMO on a subset of cardiac promoters including *Gja5* and *Pitx2* [24], [64], [65], [66], [67]. This reinforces the notion that SUMOylation does not work as an ON/OFF switch as suggested [23], but modulates transcription in more subtle ways, by responding to different signalling contexts [3]. This was elegantly shown in rod photoreceptor development where PIAS3-mediated SUMOylation of Nr2e3 caused strong transcriptional repression of cone specific genes, while the recruitment of PIAS3/Crx or PIAS3/Nr2e3 led to transcriptional activation of rod-specific promoters [68].

During the course of this project, Wang et al. described contradictory results on the effect of K51R mutation on Nkx2-5 SUMOylation and activity [23]. These authors described K51 as the sole SUMOylation site on Nkx2-5, and that mutagenesis of this site led to complete abolition of SUMO-mediated transcriptional activation together with total loss of binding to its target DNA sequences. Despite our best efforts, we could not reproduce this data, even when using the precise wild type and K51 mutant expression constructs, as supplied by this group, and identical HeLa and CV-1 cell stocks. In our hands, both wild type and mutant proteins were able to bind DNA and transactive the *Nppa* reporter in HEK293T and CV1 cells. The difference between the findings of Wang et al. and those reported here may relate to the specific methodologies used for expression of plasmids in cultured cells, or in the stringency of detection of their protein products. Importantly, however, we show here that Nkx2-5 is multiply SUMOylated in the cardiac cell line HL-1, and of the two SUMO species highlighted in in vitro studies, the unidentified slower migrating band was the predominant one. In our view, the full SUMOylation status of Nkx2-5 and the meaning of these modifications in the developing heart remains to be determined. Recently, although transgenic mice over-expressing Nkx2-5-K51R were found to be relatively normal, when crossed to mice heterozygous for a null mutation in Nkx2-5, the majority died postnatally with reduced cardiomyocyte proliferation, and atrial and ventricular septal defects [69]. These studies hint at an important role for SUMOylation of K51 in vivo, although dysregulation of Nkx2-5 as a result of the transgenic method, as seen previously for wild type Nkx2-5 [70], [71], needs to be discounted. Reduced SUMOylation of Nkx2-5 point mutations present in human CHD has also been reported. This finding suggests a correlation between SUMOylation and the loading of Nkx2-5 into functional transcriptional complexes, where it could regulate protein∶protein modifications that affect transcription factor stability and activity, and chromatin modification. For example, in mice mutant for the de-SUMOylation enzyme SENP2, the polycomb-related repressor complex PRC1 remains overactive, leading to repression of cardiac transcription factors GATA4 and GATA6 [72]. Our findings, combined with those of others, demonstrate a complex post-translational regulatory machinery underlying the expression and regulation of several of the transcription factors that function in the conserved core regulatory network for cardiogenesis. Further dissection of this machinery will have significant implications for understanding cardiac development and CHD.

## Supporting Information

Figure S1 Confirmation of the specificity of the in situ probes of SUMO pathway components. Sp6-derived sense probes display minimal background at all stages analysed when co-developed with T7-derived antisense probes in mouse embryos. All components are widely expressed in developing mouse embryos as shown in Figure 1.
(TIF)

Figure S2 SUMO-1 interacts with multiple sites in *Xenopus* Nkx2-5 but not Nkx2-2. Yeast strain Yn166 (containing a reporter gene consisting of the Gal4 UAS upstream of the β-galactosidase coding region) was co-transformed with a yeast expression vector encoding full length *Xenopus* SUMO-1 fused to the Gal4 activation domain, and a second yeast expression vector encoding the Gal4 DNA binding domain fused to the indicated fragments of *Xenopus* Nkx2-5. Amino acids contained in each mutant are indicated on the left. The domain structure of the Nkx2-5 protein is shown (top). Interaction was assessed by detection of LacZ activity. In addition, the related Nk2 homeodomain protein *Xenopus* Nkx2-2 was tested in this assay, but did not interact. In the absence of *Xenopus* SUMO-1, none of the constructs were able to activate transcription of the reporter. TN, TN domain; HD, homeodomain; NKD, NK2 specific domain; YRD, tyrosine-rich domain.
(TIF)

Figure S3 The SUMOylation banding pattern is not cell line specific. (A–B) Western blot analyses demonstrated that both HeLa (A) and COS-7 (B) cells display two SUMOylated bands using HA (SUMO-1) and Nkx2-5 antibodies. (C) Electrophoretic Mobility Shift Assay (EMSA) was performed in transiently transfected HeLa cells with the addition of 50 mM of NEM (to prevent cleaveage of the SUMO moiety). This confirms that only a small portion of the total that Nkx2-5 bound to the *Nppa* promoter is modified by SUMO conjugation, as indicated by the appearance of a supershifted band upon addition of increasing levels of anti-HA antibodies. The boxed region inset shows HA-SUMO-1 supershifted Nkx2-5 under increased contrast conditions. Note that most of the Nkx2-5 DNA-bound fraction can be specifically supershifted with Nkx2-5 antibodies.
(TIF)

Figure S4 Prediction of putative canonical SUMOylation sites by Sumoplot™ (http://www.abgent.com/tools/toSumoplot). This algorithm scored K51 and K103 as high probability sites, while K109 and K123 were predicted as low probability sites.
(TIF)

Figure S5 Enhancement of Nkx2-5 transcriptional activity by SUMO is cell line-independent. (A–B) SUMO-1 synergized with Nkx2-5 to activate the *Nppa* promoter in transient transfection of both CV-1 and COS-7 cells.
(TIF)
