# Supplementary figures and images for: Complex SUMO-1 Regulation of Cardiac Transcription Factor Nkx2-5
Source: PLoS One. 2011 Sep 12;6(9):e24812. doi: 10.1371/journal.pone.0024812 (PMC3171482; doi:10.1371/journal.pone.0024812)

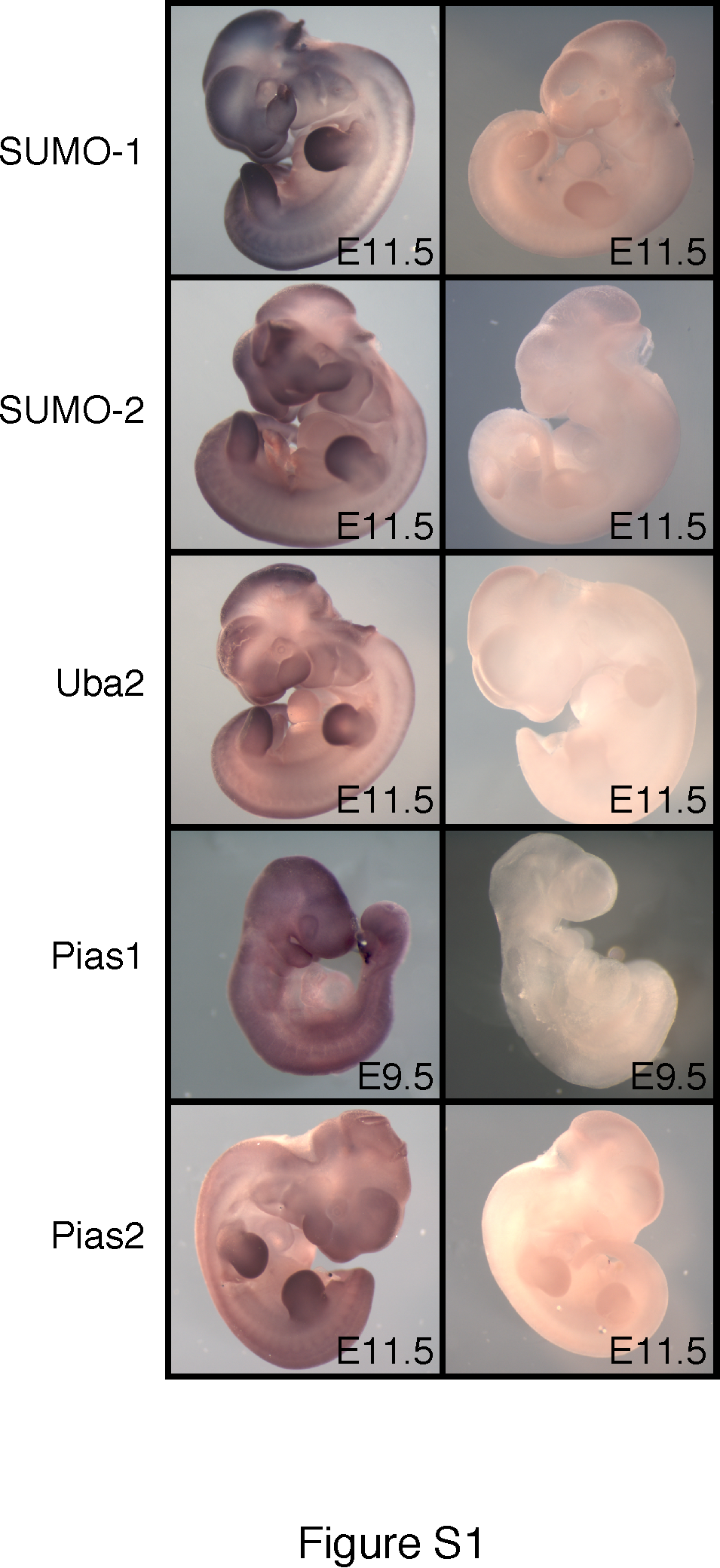

Supplement: Figure S1 — Confirmation of the specificity of the in situ probes of SUMO pathway components. Sp6-derived sense probes display minimal background at all stages analysed when co-developed with T7-derived antisense probes in mouse embryos. All components are widely expressed in developing mouse embryos as shown in Figure 1. (TIF) [file pone.0024812.s001.tif]

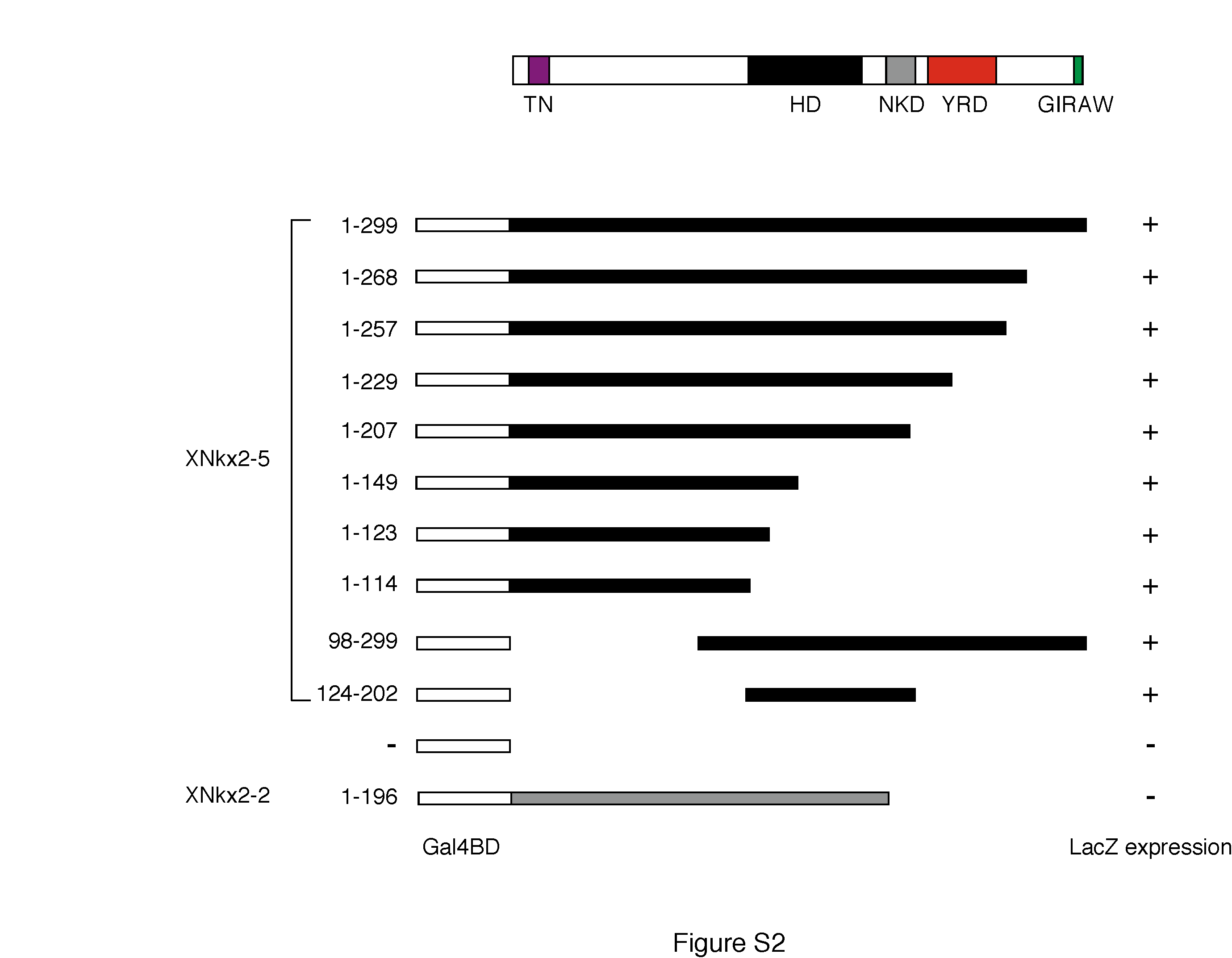

Supplement: Figure S2 — SUMO-1 interacts with multiple sites in Xenopus Nkx2-5 but not Nkx2-2. Yeast strain Yn166 (containing a reporter gene consisting of the Gal4 UAS upstream of the β-galactosidase coding region) was co-transformed with a yeast expression vector encoding full length Xenopus SUMO-1 fused to the Gal4 activation domain, and a second yeast expression vector encoding the Gal4 DNA binding domain fused to the indicated fragments of Xenopus Nkx2-5. Amino acids contained in each mutant are indicated on the left. The domain structure of the Nkx2-5 protein is shown (top). Interaction was assessed by detection of LacZ activity. In addition, the related Nk2 homeodomain protein Xenopus Nkx2-2 was tested in this assay, but did not interact. In the absence of Xenopus SUMO-1, none of the constructs were able to activate transcription of the reporter. TN, TN domain; HD, homeodomain; NKD, NK2 specific domain; YRD, tyrosine-rich domain. (TIF) [file pone.0024812.s002.tif]

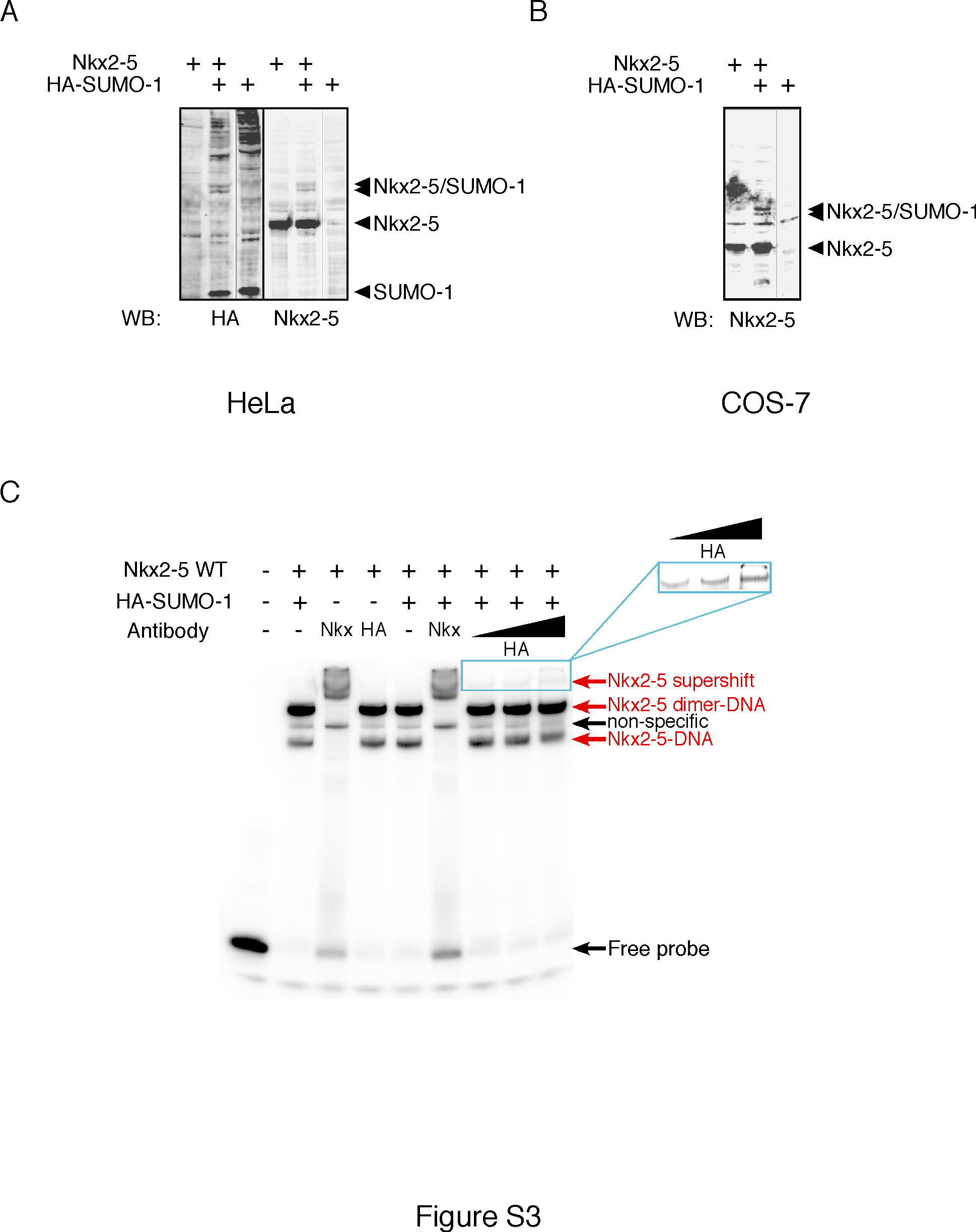

Supplement: Figure S3 — The SUMOylation banding pattern is not cell line specific. (A–B) Western blot analyses demonstrated that both HeLa (A) and COS-7 (B) cells display two SUMOylated bands using HA (SUMO-1) and Nkx2-5 antibodies. (C) Electrophoretic Mobility Shift Assay (EMSA) was performed in transiently transfected HeLa cells with the addition of 50 mM of NEM (to prevent cleaveage of the SUMO moiety). This confirms that only a small portion of the total that Nkx2-5 bound to the Nppa promoter is modified by SUMO conjugation, as indicated by the appearance of a supershifted band upon addition of increasing levels of anti-HA antibodies. The boxed region inset shows HA-SUMO-1 supershifted Nkx2-5 under increased contrast conditions. Note that most of the Nkx2-5 DNA-bound fraction can be specifically supershifted with Nkx2-5 antibodies. (TIF) [file pone.0024812.s003.tif]

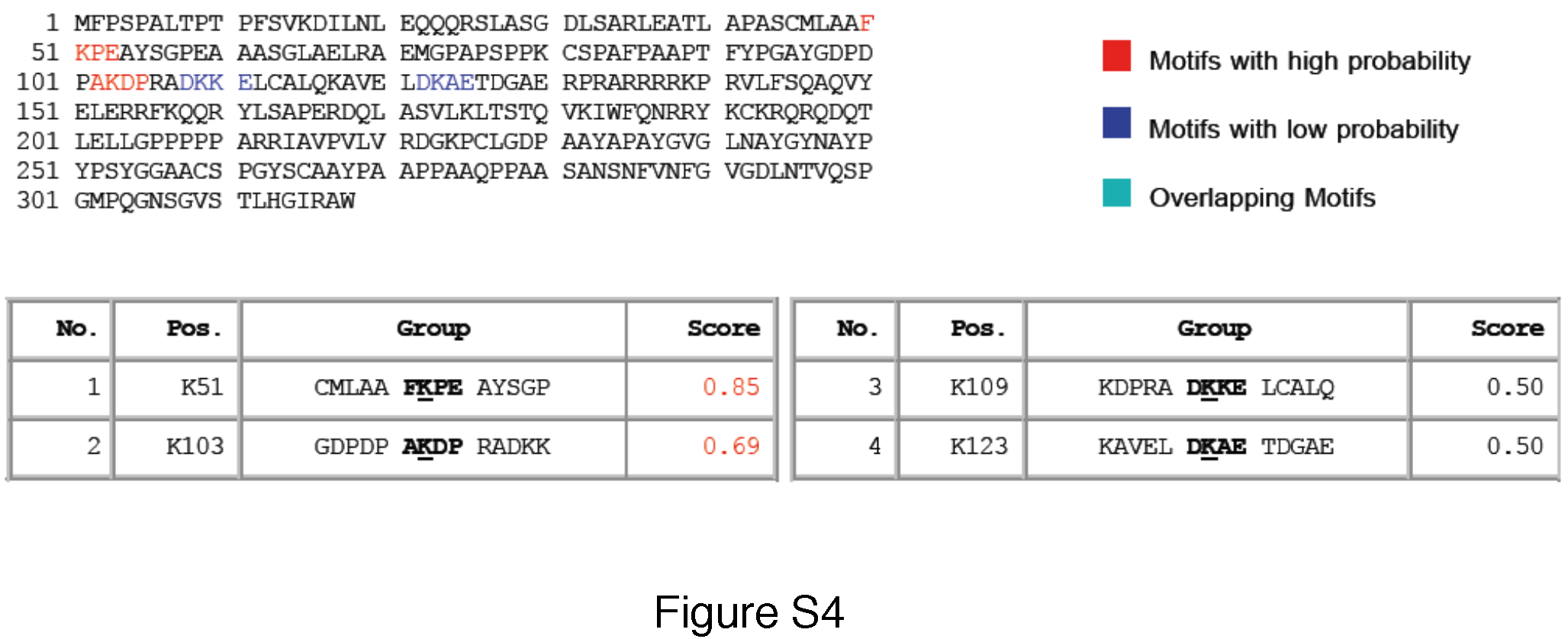

Supplement: Figure S4 — Prediction of putative canonical SUMOylation sites by Sumoplot™ (http://www.abgent.com/tools/toSumoplot). This algorithm scored K51 and K103 as high probability sites, while K109 and K123 were predicted as low probability sites. (TIF) [file pone.0024812.s004.tif]

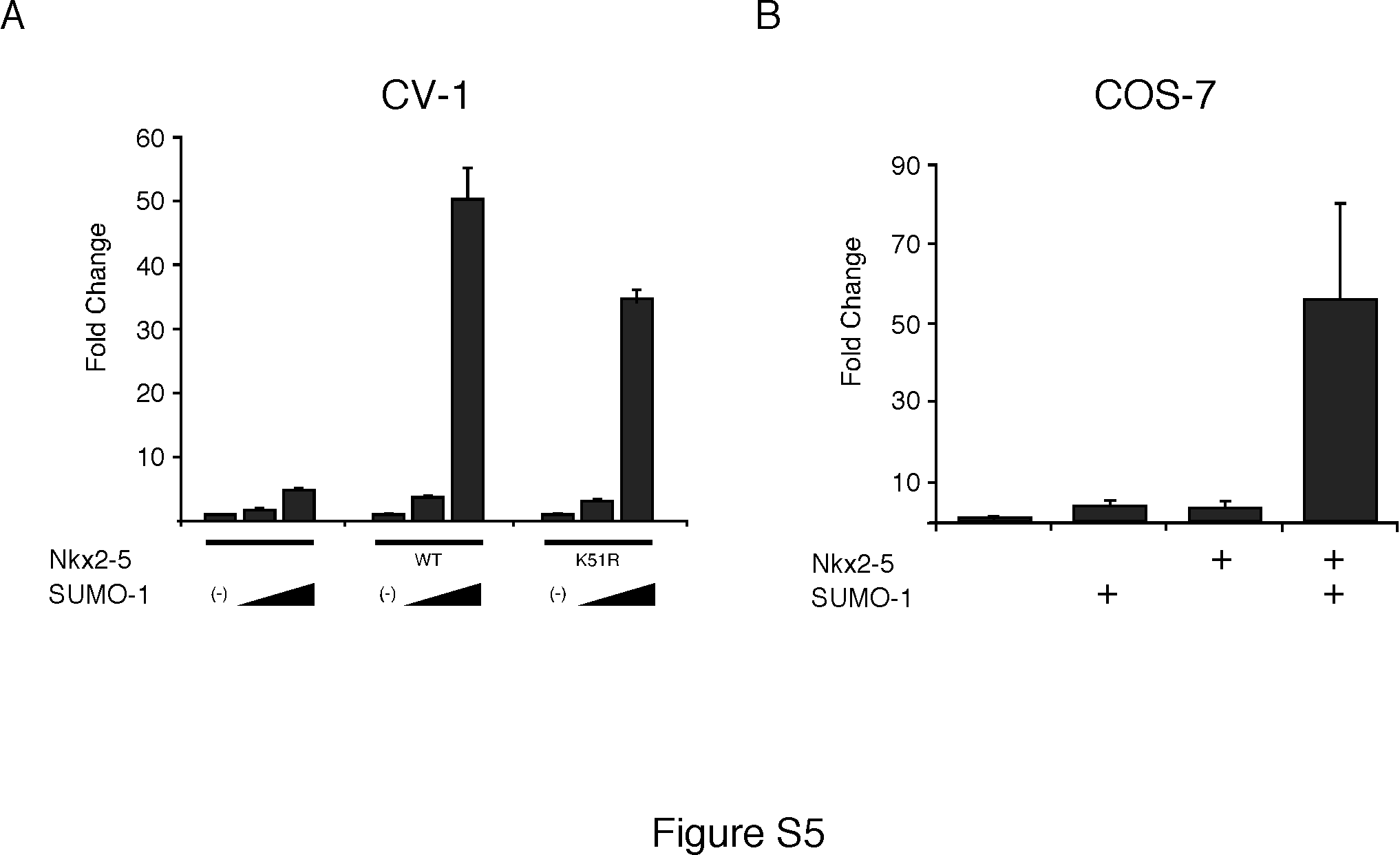

Supplement: Figure S5 — Enhancement of Nkx2-5 transcriptional activity by SUMO is cell line-independent. (A–B) SUMO-1 synergized with Nkx2-5 to activate the Nppa promoter in transient transfection of both CV-1 and COS-7 cells. (TIF) [file pone.0024812.s005.tif]
